# Hop Tannins as Multifunctional Tyrosinase Inhibitor: Structure Characterization, Inhibition Activity, and Mechanism †

**DOI:** 10.3390/antiox11040772

**Published:** 2022-04-13

**Authors:** Jiaman Liu, Yanbiao Chen, Xinxin Zhang, Jie Zheng, Weiying Hu, Bo Teng

**Affiliations:** 1 Guangdong Provincial Key Laboratory of Marine Biotechnology, Shantou University, Shantou 515063, China; 19jmliu1@stu.edu.cn; 2 Guangzhou Nali Biotechnology Co., Ltd., Guangzhou 510000, China; yanbiao.chen@hotmail.com; 3 College of Science, Shantou University, Shantou 515063, China; 19xxzhang@stu.edu.cn (X.Z.); 19jzheng1@stu.edu.cn (J.Z.); 19wyhu@stu.edu.cn (W.H.)

**Keywords:** hop tannins, polyphenols, condensed tannin, tyrosinase inhibitor, whitening agent

## Abstract

The application of hops could be extended to obtain higher commercial values. Tannins from hops were assessed for their tyrosinase inhibition ability, and the associated mechanisms were explored. Nuclear magnetic resonance (NMR) and high-performance liquid chromatography–electrospray ionization–tandem mass spectrometry (HPLC–ESI–MS/MS) revealed that the hop tannins were characterized as condensed tannins with (epi)catechin and (epi)gallocatechin as subunits and an average polymerization degree of 10.32. Tyrosinase inhibition assay indicated that hop tannins had an IC_50_ = 76.52 ± 6.56 μM. Kinetic studies of the inhibition processes indicated the tannins provided inhibition through competitive–uncompetitive mixed reactions. In silico molecule docking showed that tannins were bound to the active site of tyrosinase via hydrogen and electrovalent bonds. Circular dichroism (CD) observed the structural variation in the tyrosinase after reacting with the tannins. Fluorescence quenching analysis and free radical scavenging assays indicated that the tannins had copper ion chelating and antioxidant activities, which may also contribute to inhibition. The intracellular inhibition assay revealed that the melanin was reduced by 34.50% in B16F10 cells. These results indicate that these tannins can be applied as whitening agents in the cosmetics industry.

## 1. Introduction

Melanin is responsible for providing protection in human skin against ultraviolet radiation, stress from environmental pollutants, and toxic drugs and chemicals. However, abnormal melanin production could result in melasma, solar lentigo, freckles, acne scars, age spots, and other dermatological problems [1]. It is reported that approximately 15% of the global population invests in skin-whitening products [1]. The rate-limiting steps of overall melanogenesis are catalyzed by tyrosinase; therefore, tyrosinase has been recognized as a therapeutic target for controlling abnormal melanin synthesis [2,3,4]. Furthermore, the inhibition or reduction of melanin synthesis by a tyrosinase inhibitor has become a commonly accepted method for most of the whitening agents in the cosmetics and medical industries [5].

Consequently, many natural compounds, discovered from plants and especially food materials, have been evaluated for their tyrosinase inhibition ability. These compounds can be classified as resveratrol derivates, which were mainly found in grapes and wines, and inhibited tyrosinase through suicide substrate inhibition with limited cytotoxicity effects [6]; diphenyl compounds, with parallel benzene rings as functional groups, which exhibit an inhibition effect by forming a π–π stack with the enzyme surface [7]; indole derivates, which displayed inhibition ability by binding to either a free enzyme or to an enzyme–substrate complex [8]; and thioureas, which impacted the valence state of the active-site copper ions through a chelating reaction, inhibiting the activity of tyrosinase [9]. Other compounds from herbs and fruits, such as hydroxycinnamic acid derivates, chalcones, flavanones, peptides, etc., were proved to have tyrosinase inhibition ability [1,10]. Considering the penetration issues, previous studies mainly focused on low-molecular-weight inhibitors [11]. Since puncture outfits, such as aqua lifting and microneedle, are widely applied [12,13], high-molecular-weight natural compounds have become a new choice for inhibitors.

Hops (inflorescence of *Humulus lupulus* L.) are conventionally used in beer brewing to impart an attractive aroma and bitter taste. Hops are mainly produced in the U.S., Germany, and the Czech Republic, and their annual production has almost reached 100K tons [14]. Besides volatile oils and bitter acids, hops are also found to be rich in polyphenols with antioxidant, antimutagenic, anti-inflammatory, and antibacterial properties [15]. More importantly, the tannins, which account for almost 9% (*w*/*w*) of the hops, can be easily extracted using hot water [16]. These properties enable hop tannins to be fine natural resources for applications that might provide greater economic value [17].

In the shorter conference version of this research, we found that hop tannins showed tyrosinase inhibition ability, and preliminarily observed the tyrosinase–tannin interaction with molecular docking and circular dichroism [18]. In the current study, a more specific structural characters of hop tannins were characterized with nuclear magnetic resonance (NMR), followed by high-performance liquid chromatography–electrospray ionization–tandem mass spectrometry (HPLC–ESI–MS/MS). Then, more detailed mechanistic information was revealed through an inhibition kinetic assay. Multifunctional inhibition was evaluated on the tannin-induced tyrosinase structure variation, the copper ion chelating reaction, and antioxidant ability. Finally, intracellular tyrosinase inhibition and melanin production were assessed with B16F10 cells. The results could extend the potential application of hop tannins.

## 2. Materials and Methods

### 2.1. Preparation of Hop Tannins

Tannin extraction and purification were performed in accordance with our previous reports [19,20]. In short, fresh hops (harvested from Gansu province, China, 100°47’75.5” E, 38°96’95.7” N, identified by Dr. Jinwei Zhang (College of Life Science, Lanzhou University), referenced with herbarium (No. 00091847) stored in the Herbarium of Lanzhou University) were ground into fine powders using a plant tissue pulverizer. Then, the tannins were extracted from the powder (200 g) by using a 70% acetone–water solution (4.0 L) for 48 h at 25 °C. After completion of the extraction, the residue was removed by an ash-free paper filtration, and acetone was removed by rotary evaporation (reduced pressure, 35 °C) to obtain a crude aqueous extract. A 1 L crude aqueous extract was then extracted three times (12 h for each time, at 25 °C) with dichloromethane (1:1, v: v) to remove the lipids; then, the crude extract of the tannins was obtained after rotary evaporation (reduced pressure, 35 °C) followed by lyophilization.

Crude tannin extract (1.0 g) was dissolved in a 10 mL 50% methanol solution and then loaded onto a Sephadex LH-20 column (φ = 3.5 cm, column bed volume = 800 mL, and conditioned with a 50% methanol solution). The column was washed with a 50% methanol solution (flow rate 25 mL/min, 5.0 L eluent) to remove simple phenolics, proteins, and sugars. The tannin fraction was then collected by elution with a 70% acetone–water solution (1.0 L eluent). After removing organic solvents and water from the tannin fraction by rotary evaporation (35 °C) and lyophilization, purified tannins were obtained and used as samples for the following experiments.

### 2.2. ^13^CNMR Analysis of Hops Tannin

^13^CNMR spectra were obtained with a Bruker Ascend 400 MHz NMR Spectrometer (Bruker BioSpin, Rheinstetten, Karlsruhe, Germany). Purified tannins (30 mg) were dissolved in a 750 µL CD_4_O–D_2_O solution (1:1, v:v) and then transferred into a nuclear magnetic tube for ^13^CNMR analysis [21]. Signals were obtained under a 100.6 MHz frequency, and the spectrum was acquired under a 0.9 s acquisition interval, 1.36 s acquisition time, 20.80 μs dwell time, 24,038 Hz sweep width, and 2 s relaxation delay. The receiver gain, power level for pulse, and 90-degree high-power pulse were 203 units, 66 W, and 10.57 s, respectively.

### 2.3. HPLC–ESI–MS/MS Analysis of Subunits

To obtain subunits for the HPLC–ESI–MS/MS study, the tannins were treated with an acid–cleavage derivatization process prior to analysis. The method was referenced in our previous report [22] and slightly modified as follows: 20 mg of tannins was dissolved in 200 μL of cysteamine hydrochloride solution (50 mg/L, dissolved in methanol); then, 50 μL of 32% hydrochloride was added and heated in a 65 °C water bath (for 30 min). Afterwards, 1250 μL of sodium acetate solution (140 mM, dissolved in Milli-Q water) was added to stop the derivatization, giving a final sample aliquot with a total volume of 1.5 mL.

The HPLC–ESI–MS/MS analysis was carried out based on previous reports [14,16]. The sample aliquot (10 μL) was injected into a Synergi Hydro-RP column (150 mm × 2 mm, 4 μ, 80 Å), which was mounted onto the HPLC–ESI–MS/MS instrument (Agilent 1290II-6460, California, CA, USA). The column was eluted with mobile phase A and B composed of formic acid/water/acetonitrile (phase A = 0.3: 99.7: 0, phase B = 0.3: 59.7: 40, v: v: v). A linear gradient elution (from 0 to 90 min, 6–20% B) was used with a 0.3 mL/min flow rate. Elutes from HPLC were analyzed under the multiple-reaction monitoring (MRM) mode and recorded from m/z 100 to 500. The ESI needle potential and the ion source temperature were set at 4000 V and 350 °C, respectively, while nitrogen (10 L/min, 45 psi) and air were used as the curtain gas and nebulizer gas, respectively. Signals were collected using a positive ion scan mode with 10 units of collision energy and a 0.1-unit step size. Based on the peak areas from each compound shown on the chromatography (Supplementary Figure S1), the subunits were quantified, and the average polymerization degree was subsequently calculated in accordance with Kennedy’s report [23].

### 2.4. Tyrosinase-Inhibition Assay

The tyrosinase inhibition effects were evaluated based on Chai’s previous report [24], including (1) determining the concentration for 50% activity inhibition (IC_50_), and (2) an inhibition kinetic assay. All the reactions were undertaken in a phosphate buffer solution (PBS, 50 mM, pH = 6.8), while tyrosinase (EC 1.14.18.1, Aladdin Biochemical Technology Co., Ltd., Shanghai, China) and L-DOPA (Aladdin Biochemical Technology Co., Ltd., Shanghai, China) were chosen as the model enzyme and substrate. The mean molecular weight of the tannins was determined as 3581 Da (Supplementary Figure S2) and applied in the analysis. The analyses were briefly as follows.

#### 2.4.1. 50% Activity Inhibition

Hop tannin solutions (50 µL) of different concentrations (0, 0.25, 0.5, 0.75, 1.0, 2.0, 3.0, and 4.0 mM) were each mixed with 1 mL of L-DOPA solution (0.5 mM) and preheated at 30 °C for 10 min. Afterwards, 50 μL of tyrosinase solution (0.1 mg/mL) was added to start the reaction; then, 300 μL of the reaction solution was immediately transferred into a 96-well microtiter plate, and the absorbances (475 nm) were recorded at the beginning (0 min) and at the end (10 min) of the reaction by a microplate reader (BioTek Synergy™ HTX, VT, USA). The inhibition rate was calculated using Equation (1), and the absorbance of the tyrosinase-L-DOPA solution without tannin addition was used as a control.

Inhibition rate (%) = [(A_2_ − A_1_) − (B_2_ − B_1_)]/(A_2_ − A_1_) × 100% (1)

where A_1_ and A_2_ are the absorbance of the control solution at 0 and 10 min of the reaction, and B_1_ and B_2_ represent the absorbance of the sample at 0 and 10 min. Then, the IC_50_ (mM) of the tannins was calculated using the inhibition rate–inhibitor concentration plot (Supplementary Figure S3).

#### 2.4.2. Inhibition Kinetic Assay

The initial velocity (ΔA_475_/min) of DOPA–quinone formation was monitored (475 nm) throughout the reaction, but under different tannin concentrations (0, 0.3, 0.6, 0.9, and 1.5 mM), different enzyme concentrations (0.1, 0.2, 0.3, 0.4, and 0.5 mg/mL), as well as different DOPA concentrations (0.1, 0.2, 0.25, 0.3, and 0.5 mM). The inhibition type was evaluated according to the Lineweaver–Burk plot and the adjusted Michaelis–Menten Equation (2):

(1/V_0_) = (K_M_/V_max_) × (1/S) + (1/V_max_) (2)

where V_0_ is the corresponding initial velocity, S is the substrate concentration, and K_M_ and V_max_ are the Michaelis’s constant and maximum reaction speed, respectively, which were obtained from the vertical and horizontal intercepts from the Lineweaver–Burk plot.

The K_M_, V_max_, concentration of hop tannins (I), slope, and intercept of the Lineweaver–Burk plot were then fitted to a secondary plot through Equations (3) and (4) to obtain the inhibition constant (K_I_) and the inhibition constant in the enzyme−substrate complex (K_IS_).

Slope = K_M_/V_max_(1 + I/K_I_) (3)

Intercept = 1/V_max_(1 + I/K_IS_) (4)

### 2.5. CD Spectroscopy

The tyrosinase and tannins were dissolved in PBS (50 mM) to obtain sample solutions with a constant tyrosinase concentration (0.4 mg/mL) but different tannin concentrations (0, 5, and 10 μM). After a 30 min (37 °C) reaction, the sample solutions were transferred into a quartz sample cell (0.1 cm path length) mounted onto a MOS-450 CD spectropolarimeter (Bio-Logic, Claix, France) [25]. The spectra (from 190 to 260 nm) were obtained with a 50 nm/min scanning rate and a 0.25 s response rate. Afterwards, the spectra (Supplementary Figure S4) were analyzed with the CDNN CD spectral deconvolution software package (version 2.1, Applied Photophysics Ltd., Leatherhead, UK) to calculate the relative content of the secondary structures of tyrosinase.

### 2.6. In Silico Molecule Docking

Molecule dockings were performed using an open-source AutoDock Vina software package (DeLano Scientific LLC, Palo Alto, CA, USA). The crystallographic structure of the tyrosinase (obtained by X-ray diffusion, PDB: 2Y9X) was taken from RCSB Protein Data Bank and used as the initial protein model. The model was then subjected to water and tropolone removal, followed by polar hydrogen addition, a missing atom correction, and a Gasteiger charge assignment. Subunits of the tannins, including catechin, epicatechin, gallocatechin, and epigallocatechin, were constructed with Chem Draw 17.0 software (Cambridge, UK) and geometrically optimized with an MM_2_ force field to obtain their preferential conformations.

Referencing Heitz’s research [26], molecular docking was performed through a blind docking simulation within a grid box (30 × 30 × 30, 0.1 nm grid spacing) placed on the geometric center of the active site (x, y, z: −7.392, −24.898, −39.626). A semiflexible docking mode, which was employed under a 10-unit energy range and 20 units of exhaustiveness, was chosen for all subunit–tyrosinase complexes. The preferential docking conformations were obtained through a sophisticated gradient optimization method, and the docking score was calculated with an empirical scoring function. Binding energies were predicted from 20 subunit–tyrosinase complexes with the lowest energies. Afterwards, the complex with the lowest energy was analyzed with PyMOL 2.2 software (Schrödinger Inc., New York, NY, USA) to observe the binding pose, related amino acid residuals, and formed hydrogen bonds.

### 2.7. Antioxidant Ability Evaluation

The antioxidant activity of the tannins was assessed through 3-ethylbenzthiazolin-6-sulfonic acid (ABTS) and 2,2-diphenyl-1-picrylhydrazyl (DPPH) radical scavenging analyses. The ABTS assay was conducted following the method described previously [27,28] and modified as follows. ABTS solution (7 mM) and potassium persulfate solution (2.45 mM) were mixed at equal volumes and stood for 12 h (at 25 °C, without light exposure) to prepare the ABTS working solution. Hop tannin solutions (0.1 mL) of different concentrations (0, 0.02, 0.04, 0.06, 0.08, 0.10, and 0.12 mM) were each mixed with 3.9 mL ABTS working solution and reacted at 25 °C for 6 min. Then, the absorption of the reaction solutions was recorded at 734 nm by the microplate reader, and samples without tannin addition were used as controls.

For the DPPH assay, the procedure was carried out following a previously described method [29]. In short, 0.1 mL hop tannin solutions of different concentrations (0, 0.02, 0.04, 0.06, 0.08, 0.10, and 0.12 mM, dissolved in methanol) were each mixed with 3 mL of DPPH solution (25 mg/L). After a 30 min reaction (25 °C), the absorbance of 300 μL of the mixtures was measured at 517 nm with the microplate reader. Free radical elimination rates were fitted with their corresponding tannin concentrations to obtain the 50% free radical elimination rate (IC_50_).

### 2.8. Fluorescence Quenching Analysis

The copper ion chelating ability of the tannins was measured by fluorescence quenching analysis. The protocol was conducted in accordance with published research [24] and adjusted as follows. Hop tannins solution (900 μL, 3 mg/mL) was reacted with 100 μL copper sulphate solution of different concentrations (0, 20, 40, 60, 80, 100 μM). The reactions proceeded at 25 °C for 1 min prior to analysis; then, solutions were transferred into the 1 cm quartz cuvette mounted onto a F-7000 fluorescence spectrometer (Hitachi, Japan). The spectra were recorded from 300 to 450 nm with a 280 nm excitation wavelength, 240 nm/min scan speed, 5 nm emission slid width, and 2.5 nm excitation slit width.

### 2.9. Intracellular Tyrosinase Inhibition and Melanin Production

#### 2.9.1. Cell Line and Cell Culture

B16F10 melanoma cells (accession number: RRID: CVCL_0159; acquired from Procell Life Science & Technology Co. Ltd., Wuhan, China) were seeded and incubated in a cell culture bottle filled with Dulbecco’s modified eagle medium (DMEM) (basic DMEM mixed with heat-inactivated fetal bovine serum (10%, volume based), penicillin (100 U/mL), and streptomycin (100 µg/mL)) for 3 days in a 37 °C incubator filled with 5% CO_2_ atmosphere. After incubation, the cell pellet was washed with PBS (10 mM) and dispersed by pipetting with 0.25% trypsin (1 mL) and harvested for cytotoxicity, cellular tyrosinase inhibition, and cellular melanin production analysis.

#### 2.9.2. Intracellular Tyrosinase-Inhibition Assay

The cellular tyrosinase inhibition was measured as described in the report from Si et al. [30]. B16F10 cells (2 mL, 1 × 10^6^ cell intensity) were seeded into a 6-well plate and incubated for 12 h. Then, the supernatant was replaced with 2 mL DMEM media with different hop tannin concentrations (0, 2.5, 5, 7.5, 10 μM) and then incubated for 48 h. After incubation, the medium was removed and the cell pellet was washed three times with PBS solution; the pellet was then dispersed with 500 μL of PBS buffer (pH 6.8, containing 1% Triton X-100) and treated with a freeze/thaw (−80 °C, 30 min/25 °C, 30 min) process following a centrifuge (12,000 rpm, 30 min) to obtain the dissolved intercellular substance. The dissolved intercellular substance (20 μL) was reacted with 180 μL of L-DOPA solution (1 mM) in a 96-well plate. After a 30 min reaction (at 37 °C), its absorption was recorded at 475 nm and the cellular tyrosinase inhibition rates were calculated using Equation (5), while cells cultured without tannin addition (hop tannin concentration = 0 μM) were used as a control.

Cellular tyrosinase-inhibition rate (%) = (D_0_ − D_n_)/D_0_ × 100% (5)

where D_n_ is the absorbance obtained from samples and D_0_ is the absorption of the sample prepared without tannin addition.

#### 2.9.3. Intracellular Melanin Inhibition Assay

B16F10 cells (2 mL, 1 × 10^6^ cell intensity) were transferred into a 6-well plate and incubated for 12 h [30]. Afterwards, the media was replaced by 2 mL DMEM media with different hop tannin concentrations (2.5, 5, 7.5, 10 μM) and incubated for 48 h. After incubation, the pellet was washed three times with PBS buffer and then dispersed with 1 mL of NaOH solution (1 N, dissolved in a water–dimethyl sulfoxide solution, 9:1, v:v). The pellet-dispersed solution was then heated at 80 °C for 1 h and the absorption was measured at 405 nm. The relative melanin production was calculated using Equation (6) and cells cultured without tannin addition were used as a control.

**Figure 1:**
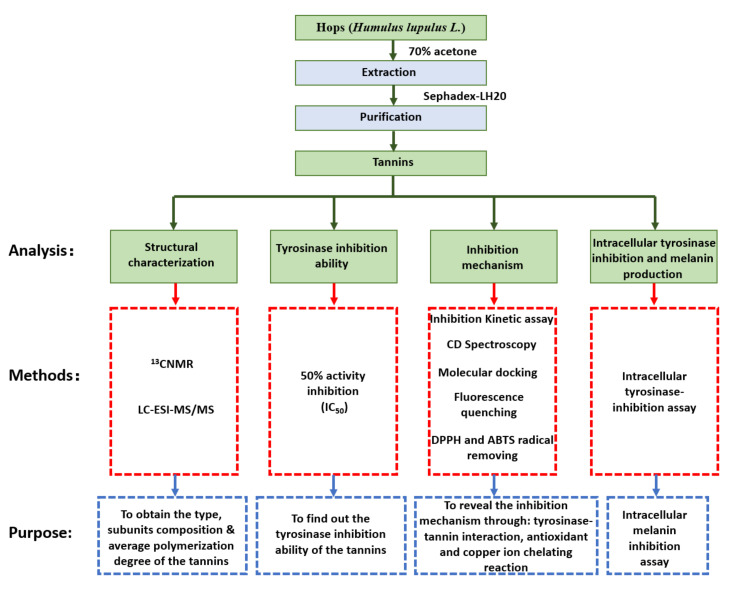
Schematic diagram of sample preparation and analyses in the study.

Intracellular melanin inhibition rate (%) = (E_0_ − E_n_)/E_0_ × 100% (6)

where E_n_ is the absorbance obtained from the samples and E_0_ is the absorption of the sample prepared without tannin addition. The schematic diagram of the study is presented in Figure 1.

## 3. Results and Discussion

### 3.1. Structural Characters of Hops Tannin

The structural characters of tannins, especially subunit composition, average molecular weight, and polymerization degree, are believed to be crucial for their chemical and biochemical properties [19,20,31]. Therefore, the structural characters of hop tannins were analyzed by ^13^CNMR and HPLC–ESI–MS/MS.

**Figure 2:**
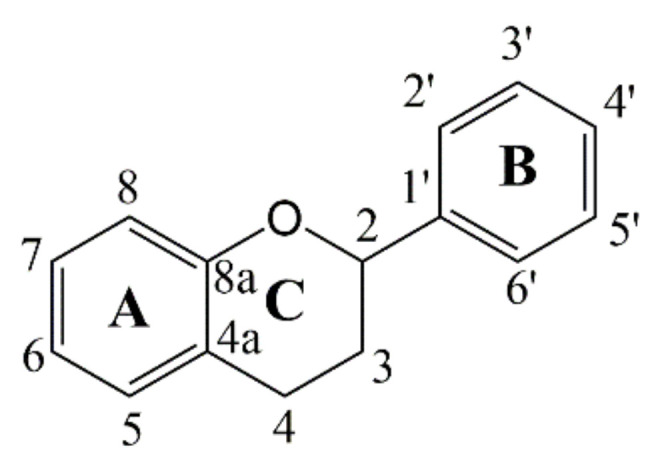
Structure and numbering scheme of the flavanol-3-ol subunit.

The ^13^CNMR spectra (Supplementary Figure S5) indicated that the tannins can be classified as polyflavonoids (condensed tannins) due to the absence of signals from carbonyl groups and glucose moieties (typical structural features of a hydrolyzable tannin) [19]. Consequently, the resonances from ^13^CNMR spectra were assigned to the carbons from the A-ring, the B-ring, and the pyrenoid C-ring of the flavanol-3-ol subunits (Figure 2).

Resonances from 70 to 90 ppm, which are sensitive to the pyrenoid carbons on the B-ring, could provide stereochemistry information [32]. With reference to previous studies [33], the resonances appearing at 77.2 and 73.1 ppm were assigned to C-2 on cis and trans stereoisomers (Table 1), while the resonance lines at 70.7 and 66.7 ppm were attributed to C-3 on terminal and extending subunits, respectively. Based on Czochanska’s report [32], chemical shifts appearing at 156.7, 102.3, 97.9, 107.6, and 115.1 ppm were attributed to the phenolic carbons on the A-ring, while resonances at 132.6, 115.3, 145.4, 145.9, 116.2, and 119.4 ppm were attributed to the phenolic C-ring. The ^13^CNMR results confirmed that these tannins were a condensed type composed of flavanol-3-ol subunits with different stereoisomers. To obtain more detailed information of these subunits, we conducted HPLC–ESI–MS/MS for further characterization and quantification.

**Figure 3:**
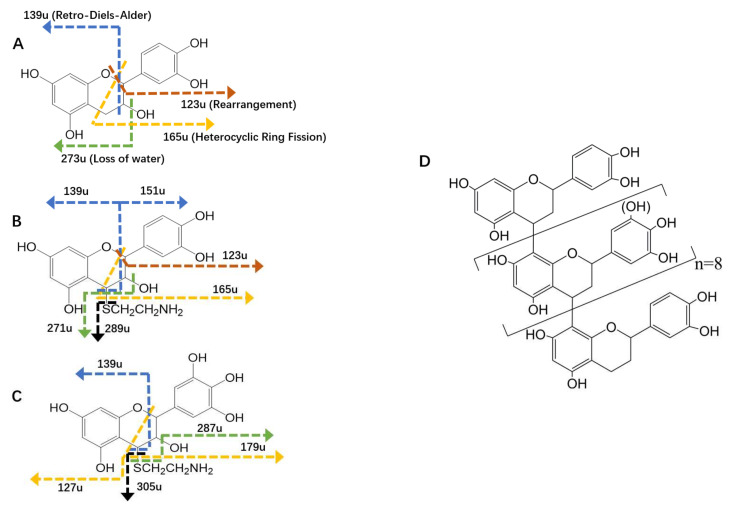
The proposed structure of the (epi)catechin (**A**), (epi)catechin–cysteamine (**B**), and (epi)gallocatechin (**C**) released from hop tannins (**D**) after acid–cleavage reaction. The fragmentation pathways are illustrated: blue lines = RDA reaction; brick-red lines = rearrangement of B-ring; yellow lines = HRF reaction; green lines = loss of water; black lines = loss of cysteamine adduct.

Subunits and their derivates with different retention times were found through HPLC chromatography (Supplementary Figure S1). After ESI–MS/MS analysis (Supplementary Figure S6), different fragmentation pathways of the typical flavanol-3-ols were shown, including the retro-Diels–Alder (RDA) reaction of the pyrenoid C-ring, the heterocyclic ring fission (HRF) of the C-ring, and the rearrangement of the B-ring (Figure 3A–C) [34,35].

The fragment ions at 139 u (Table 2), which corresponded to the loss of a neutral fragment containing the B-ring, through the RDA reaction [35] (Figure 3), were shown on the spectra of all subunits. These fragment ions indicated the existence of phloroglucinol A-rings on their structures. This result was further enhanced by the fragment ions at 127 u, 165 u, and 179 u, which also resulted from the loss of the phloroglucinol A-ring, but through HRF reactions [35]. The 123 u were generated by the rearrangement of the pyrenoid C-ring after the cleavage of the two covalent bonds at the C-2 position, while 273 u were attributed to the loss of water molecules (18 u), as commonly seen in previous studies [34,36].

The abundant fragment ions at 289 u and 305 u served to illustrate the loss of the cysteamine moiety (77 u), while 271 u and 287 u were attributed to the loss of both water and cysteamine moieties (77 + 18 u). These fragment ions demonstrated that the related subunits were released from the extension position of the tannins [22].

In general, the subunits and their derivates, including (epi)catechin (291 u), (epi)catechin–cysteamine (366 u), and (epi)gallocatechin–cysteamine (382 u), were identified. The relative contents of these subunits were 8.83%, 78.76%, and 12.40%, respectively. Furthermore, hop tannins were found to have (epi)catechin as a terminal unit, and (epi)catechin and (epi)gallocatechin as extension units. This structural character is in agreement with the findings of Kennedy et al. [23]. Based on the content of the terminal and extension subunits, and referencing previous literature [14], the average polymerization degree of the tannins was calculated as 10.32. Our initial conference version of this study did not address the specific molecular structure of the hop tannins [18]. In accordance with the above information, typical structures of the tannins were accordingly speculated and are shown in Figure 3D.

### 3.2. Tyrosinase Inhibition Ability of Hop Tannins

The tyrosinase inhibition ability of hop tannins was evaluated and expressed as IC_50_. Kojic acid, a famous skin-whitening agent [37], was also tested for tyrosinase inhibition and used as a comparison.

The results (Supplementary Figure S3) implied that the tannins showed an IC_50_ value of 76.52 ± 6.56 μM, which is comparable to kojic acid (49.54 ± 2.08 μM). Kojic acid has previously been proven to have stronger tyrosinase inhibition ability than many other commercial inhibitors, such as arbutin, ascorbic acid, and phloroglucinol [38]. These results also indicate that the tannins may also have better or comparable inhibition ability as those commercial inhibitors. To answer why the tannins showed such strong tyrosinase inhibition ability, the inhibition mechanism was explored and is discussed in the following sections.

### 3.3. Inhibition Mechanism Exhibited by Hops Tannin

**Figure 4:**
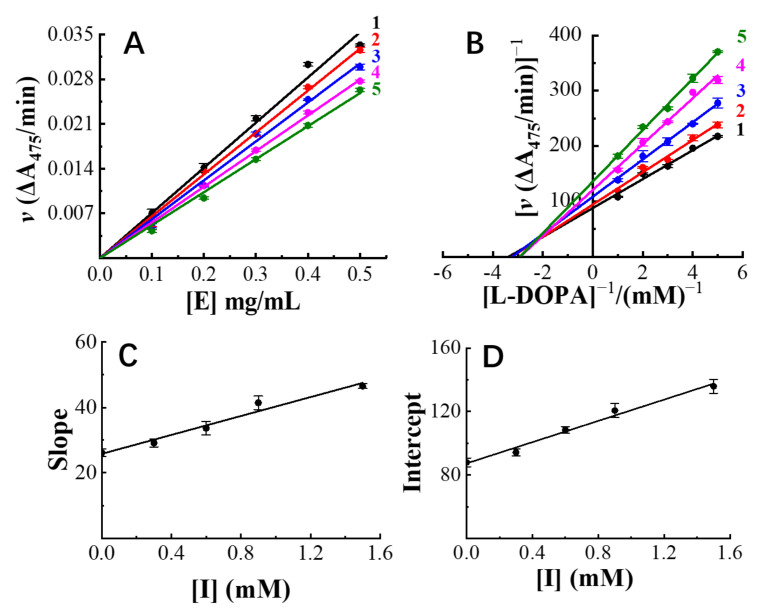
Tyrosinase concentration–reaction rate plots (**A**) and Lineweaver–Burk plots (**B**) of hop tannins of different concentrations (from 1 to 5, tannin concentration: 0, 0.041, 0.027, 0.041, and 0.068 mM); the plot of slope (**C**) or intercept (**D**) versus hop tannin concentration for determining inhibition constants K_I_ and K_IS_.

After fitting the initial velocities with their corresponding tyrosinase concentrations, the tyrosinase concentration–reaction rate plots were obtained and are presented in Figure 4A. All fitted curves were observed passing through the origin of the axis, despite increasing the tannin concentrations. This graph not only indicates that the catalyzed reaction will not stop unless tyrosinase is completely eliminated ([E] = 0 mg/mL), but also that it is unique for its reversible inhibition [39].

The Lineweaver–Burk plots (Figure 4B), which were obtained from the adjusted Michaelis–Menten equation, showed different y-axis intersects with varying tannin concentrations. Moreover, all the fitted velocity–substrate concentration plots were observed as intersecting in the second quadrant. Based on Whiteley’s research [39], these graphs not only indicate that the maximum reaction speed and Michaelis’s constant (K_M_) were both affected by changing inhibitor concentrations, but more importantly, they imply that tannins inhibited tyrosinase activity in a competitive–uncompetitive mixed way. In other words, hop tannins showed inhibition ability through binding on either active or nonactive sites of tyrosinase.

One of the most important mechanisms for competitive inhibition is the structural similarity between inhibitors and substrates [5]. L-DOPA, which is the substrate of the current reaction, has catechol as a functional moiety [40,41]. The tannins are dominantly composed of (epi)catechin as subunits and are rich in catechol moieties located on their terminal and extension positions. It is highly possible that the observed competitive inhibition could be attributed to these abundant catechol moieties on hops tannin. In addition, the tannins were able to bind with proteins by forming hydrogen bonds and hydrophobic forces [42], which might be the reason for the uncompetitive inhibition observed in the current study. The dissociation constants of the inhibitor–tyrosinase complex (K_I_) and of the inhibitor–tyrosinase–substrate complex (K_IS_) were obtained through slopes of the secondary fitted lines (Figure 4C,D). As expected, tannins showed a K_I_ value of 0.081 ± 0.005, close to the K_IS_ value (0.119 ± 0.007). This indicates that the hops tannin–tyrosinase complex showed similar binding forces to the hops tannin–L-DOPA–tyrosinase complex [43], which also highlights the possibility of binding on both active and nonactive sites of tyrosinase.

The secondary structures of tyrosinase, before and after binding with tannins, were observed through CD spectra, and are detailed in Table 3. The tyrosinase showed secondary structures in terms of a 61.85% α-helix, a 7.87% β-sheet, a 16.71% β-turn, and a 13.57% random coil. After reacting with 5 μM of tannins, the contents of the α-helix, β-sheet, β-turn, and random coil were changed to 65.20%, 6.89%, 16.61%, and 11.30%, respectively. This result can be attributed to the binding-induced protein structural variation, which is commonly seen in tannin–protein interactions [44]. This result was further strengthened after more hop tannin molecules became involved in the binding reaction. The contents of the secondary structures, especially α-helix and random coil, were observed to change to higher/lower levels when 10 μM of hop tannins was added to the tyrosinase solution. This result implies that the tannins are able to affect the secondary structure of the tyrosinase and hints that the tannins have the potential to inhibit tyrosinase by binding to the nonactive sites.

**Figure 5:**
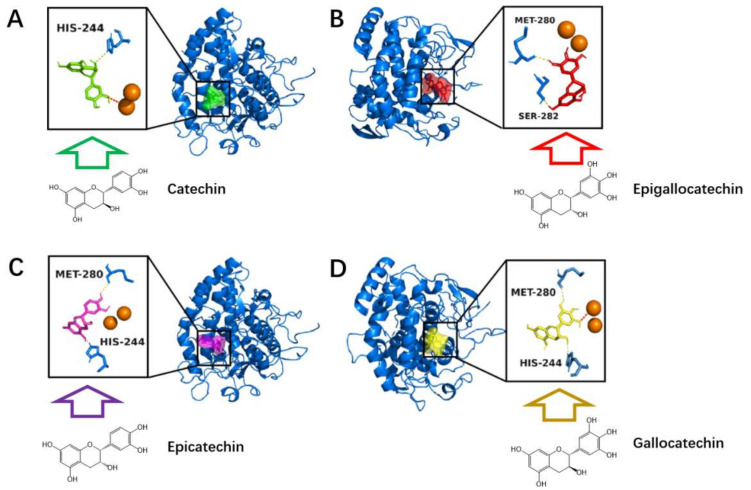
Molecular docking of catechin (**A**), gallocatechin (**B**), epicatechin (**C**), and epigallocatechin (**D**) on active site of tyrosinase.

To provide insight into the tyrosinase inhibition, molecular docking was performed. The subunits, including catechin, epicatechin, gallocatechin, and epigallocatechin, were chosen as ligand models and were applied for the docking. The complexes with the lowest energies are shown in Figure 5.

The subunits were all observed as embedding into the active site and binding with tyrosinase by forming hydrogen bonds with HIS-244, SER-282, and MET-280 (Figure 5). These results illustrate the active site bindings obtained from the inhibition kinetic analysis. Furthermore, molecular docking also discovered the interaction between copper ions and subunits. The electrovalent bonds were observed while catechin and epigallocatechin were introduced into the active site. The tyrosinase catalyzed oxidation of these model compounds were observed through the UV-Vis spectrum (Supplementary Figure S7), confirming that these model compounds interacted with tyrosinase through the active site. To obtain more information on the observed copper ion–tannin reaction, the hop tannin was analyzed for its copper ion chelating ability, as shown below.

### 3.4. Copper-Chelating and Antioxidant Abilities

Many compounds with copper chelating ability and antioxidant activities were identified to have tyrosinase inhibition activity, not only because the oxidation state of copper ions embedded into the active site plays a key role in the tyrosinase-catalyzed reactions, but also because the L-DOPA to DOPA–quinone transition is basically an oxidation reaction [1].

**Figure 6:**
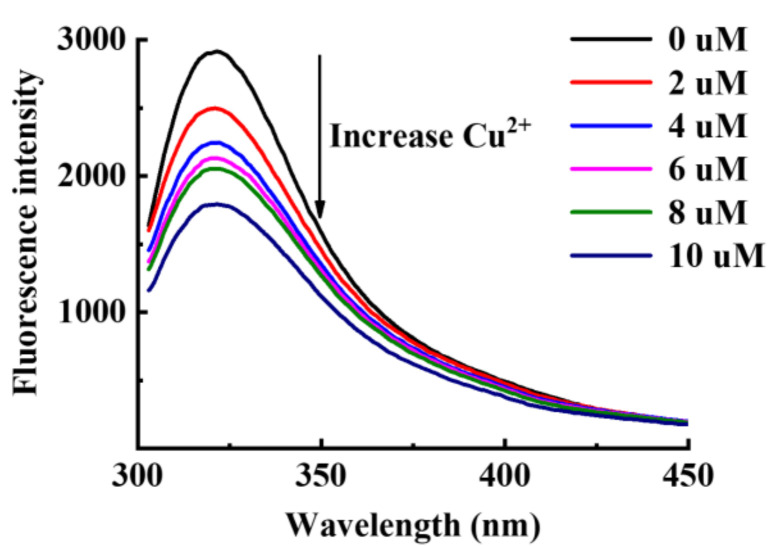
Fluorescence emission spectra of hop tannin solutions with different Cu^2+^ concentrations.

The Cu^2+^ chelating ability of hop tannins was assessed through fluorescence analysis and is shown in Figure 6. The fluorescence of hop tannins was observed to reduce with increasing Cu^2+^ concentrations in the solution. This phenomenon can be attributed to the fluorescence quenching induced by the Cu^2+^ chelation. The fluorescence emissions of the tannins are believed to be from benzene rings with hydroxyl groups [24]. The abundant hydroxyl groups on their molecules enable the tannins to react with most of the metal ions through chelation to form complexes [45]. The hop tannins–tyrosinase interaction-induced fluorescence quenching was not addressed in our previous study [18]. In the current study, the copper ion chelating ability was also observed through fluorescence and molecular docking analysis further enhanced the observation.

The antioxidant activity of tannins was evaluated through its DPPH^·^ and ABTS^·+^ radical scavenging abilities in comparison with a known antioxidant (ascorbic acid) and a tyrosinase inhibitor (hydroquinone) (Table 4). Hop tannins provided significantly lower IC_50_ values than ascorbic acid, indicating a substantial antioxidant capability that may also contribute to the tyrosinase inhibition ability, as previously noted for ascorbic acid [46]. Compared with hydroquinone, tannins showed better ABTS^·+^ removing ability and similar DPPH^·^ scavenging ability, indicating a considerable antioxidant ability of hops tannin.

In general, hop tannins presented outstanding tyrosinase inhibition ability, not only via binding on the active or nonactive sites of tyrosinase, but also by affecting the tyrosinase structure, and by its copper ion chelating ability and antioxidant activity.

### 3.5. Cell Viability, Intracellular Tyrosinase Inhibition, and Melanin Production

**Figure 7:**
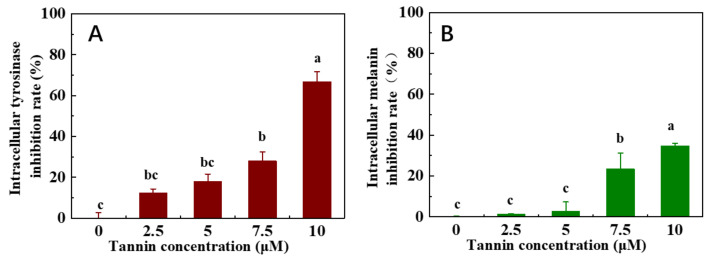
Intracellular tyrosinase inhibition rate (**A**) and intracellular melanin inhibition rate (**B**) of hop tannins.

The intracellular tyrosinase and the corresponding melanin inhibition ability of hop tannins are evaluated and presented in Figure 7. The intracellular tyrosinase inhibition rates and the intracellular melanin inhibition rates were all enhanced by increasing tannin concentrations (Figure 7A,B). This finding demonstrates that hop tannins are not only able to inhibit tyrosinase in solutions, but also regulated tyrosinase and subsequently suppressed melanin synthesis in B16F10 cells. Furthermore, a 66.67% tyrosinase inhibition rate as well as a 34.65% melanin inhibition rate were observed when 10 μM of tannins was used for inhibition.

## 4. Conclusions

Hop tannins are composed of (epi)catechin as a terminal subunit and (epi)catechin and (epi)gallocatechin as extension subunits, and have an average polymerization degree of 10.32. They present the multifunctional tyrosinase inhibition effects of binding onto the active site and forming a hydrogen bond with tyrosinase; chelating with copper ions, thereby influencing the valence state of the ions; affecting the structure of tyrosinase; and presenting antioxidant abilities during an enzyme catalysis reaction. As a consequence, the tannins show a competitive–uncompetitive mixed inhibition to tyrosinase. Hop tannins also presented intracellular tyrosinase and melanin inhibition abilities, a 66.67% tyrosinase inhibition rate, and a 34.65% melanin inhibition rate when 10 μM of tannins was used. These results demonstrate that hop tannins may have the potential to be applied as a whitening agent in the cosmetics industry.

## Figures and Tables

**Table 1:** ^13^CNMR resonance assignments of hops tannin.

A-Ring (Phenolic Carbons)					
C-5, C-7	C-6	C-8	C-4a	C-8a	
156.7	102.3	97.9	107.6	155.1	
**B-ring (phenolic carbons)**					
C-1′	C-2′	C-3′	C-4′	C-5′	C-6′
132.6	115.3	145.4	145.9	116.2	119.4
**C-ring (pyrenoid carbons)**					
C-2 (*trans*)	C-2 (*cis*)	C-3 (extension)	C-3 (terminal)	C-4	
77.2	73.1	70.7	66.7	56.0	

**Table 2:** Fragment ions obtained from subunits of hops tannin.

Precursor Ions	Retention Time (min)	Fragment Ions	Proposed Structure
291	29.0	273, 165, 139, 123	(epi) catechin
	51.3		
366	20.5	289, 271, 165, 151, 139, 123	(epi) catechin-cysteamine
	35.5		
382	9.9	305, 287, 179, 139, 127	(epi) gallocatechin-cysteamine
	22.8		

**Table 3:** Relative content of the secondary structure after reaction with tannins.

Concentration of Hop Tannins	α-Helix	β-Sheet	β-Turn	Random Coil
0	61.85	7.87	16.71	13.57
5 μM	65.20	6.89	16.61	11.30
10 μM	67.23	6.91	16.78	9.18

**Table 4:** Free radical scavenging abilities of hops tannin ^1^.

	DPPH^·^ (IC_50_ μM)	ABTS^·+^ (IC_50_ μM)
Hops tannin	1.17 ± 0.08 ^b^	1.52 ± 0.02 ^b^
Ascorbic acid	22.04 ± 0.28 ^a^	17.83 ± 0.30 ^a^

^1^ Data are expressed as the mean of three replicates ± standard deviation; the data were compared by one-way ANOVA and Tukey’s post hoc test; different letters within a column indicate a significant difference, *p* < 0.05.

## Data Availability

All data generated or analyzed during this study are included in this published article and its Supplementary Materials.

## Supplementary Materials

The following supporting information can be downloaded at: https://www.mdpi.com/article/10.3390/antiox11040772/s1, Figure S1: The chromatography of the hop tannin subunits after acid–cleavage treatment; Figure S2: GPC chromatography of the hops tannin; Figure S3: Inhibition rates and corresponding hop tannin and kojic acid concentrations fitted curves for calculate the 50% activity inhibition (IC_50_); Figure S4: Circular dichroism spectrum of tyrosinase after react with hop tannins with different concentrations; Figure S5: The ^13^CNMR spectra of the hop tannins with assignment of each carbons; Figure S6: The spectrum of the subunits obtained by ESI-MS/MS analysis; Figure S7: UV-Vis spectra of the catechin, epicatechin, gallocatechin, and epigallocatechin before and after reacting with tyrosinase.
