# Supplementary material for: Hop Tannins as Multifunctional Tyrosinase Inhibitor: Structure Characterization, Inhibition Activity, and Mechanism
Source: Antioxidants (Basel). 2022 Apr 13;11(4):772. doi: 10.3390/antiox11040772 (PMC9027561; doi:10.3390/antiox11040772)
Supplement: Supplementary file 1 [file antioxidants-11-00772-s001.zip › antioxidants-1630385-supplementary.pdf]

**Supplementary information 1**

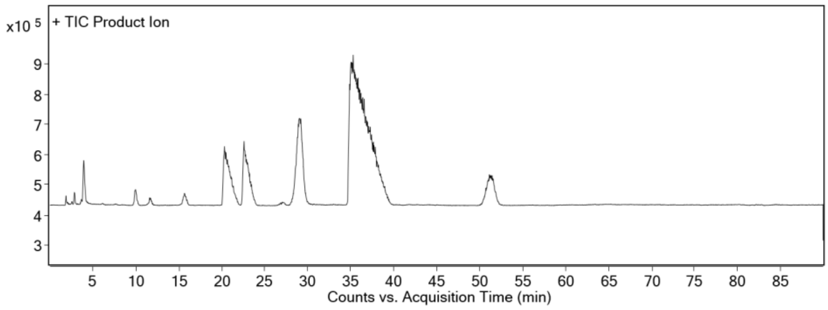


Figure S1. The TIC chromatography of the hops tannin subunits after acid-cleavage treatment.

**Supplementary information 2**

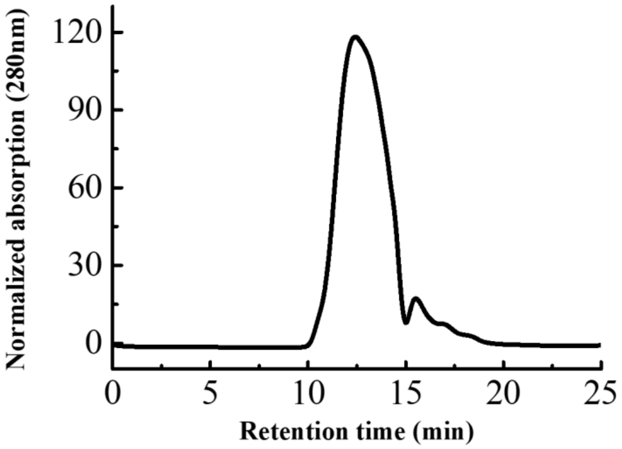


Figure S2. Gel-permeation chromatography of the hops tannin. The analysis was provided in accordance with our previous research (Teng Bo, Hayasaka Yoji, Paul A. Smith and Keren Bindon. Effect of grape seed and skin tannin molecular mass and composition on the rate of reaction with anthocyanin and subsequent formation of polymeric pigments in the presence of acetaldehyde. Journal of Agricultural and food chemistry, 2019, 67, 8938-8949.). The average molecular mass of the hops tannin was determined as 3581 Da while grape skin tannins (condensed type, same as hops tannin) with different molecular weights were used as standard sample for calculation.

**Supplementary information 3**

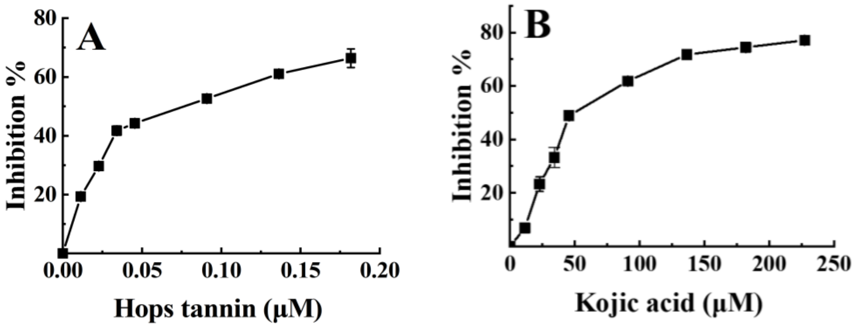


Figure S3. Inhibition rates and corresponding hops tannin (A) and kojic acid (B) concentrations fitted curves for calculate the 50% activity inhibition ($IC_{50}$).

Table S1. Tyrosinase-inhibition ability of hops tannin and kojic acid. Data were expressed as the mean of three replicates ± standard deviation, and the data were compared by one-way ANOVA and a Tukey's post hoc test, where different letters within a column indicate a significant difference where $P<0.05$. $IC_{50}$ was the concentration of inhibitor with half maximal inhibitory effect; data were expressed as the mean of 3 replicates ± standard deviation. The data were compared by one-way ANOVA and a Duncan's post hoc multiple comparison, where different letters within a column indicate a significant difference.

| | Hops tannin | Kojic acid |
|---|---|---|
| $IC_{50}$ (μM) | 76.52 ± 6.56 [a] | 49.54 ± 2.08 [b] |

**Supplementary information 4**

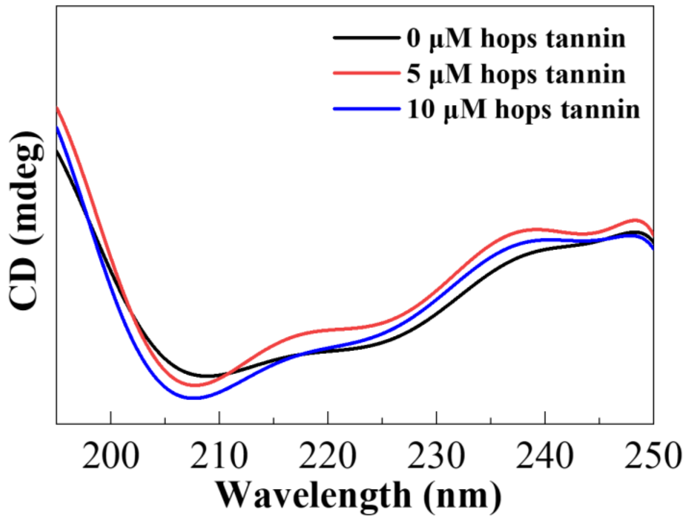


Figure S4. Circular dichroism spectrum of tyrosinase after react with hops tannin with different concentrations.

**Supplementary information 5**

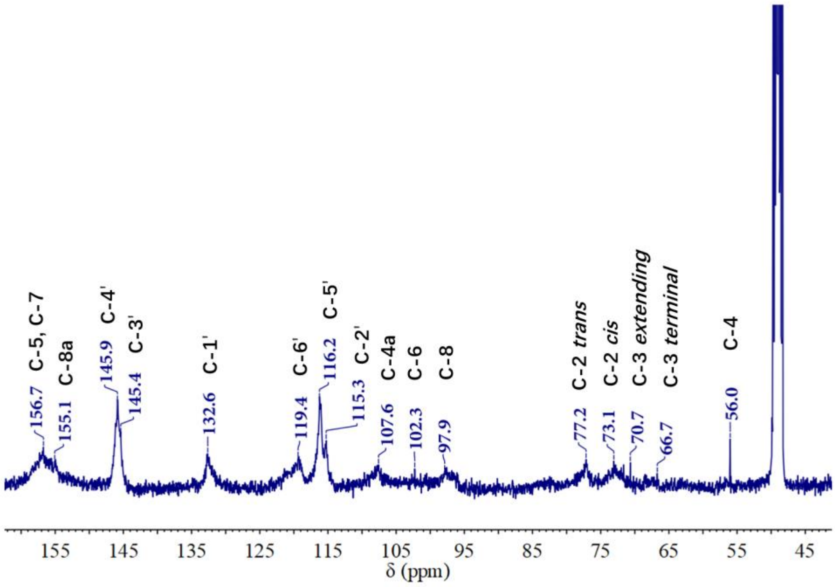


Figure S5. The $^{13}$CNMR spectra of the hop tannins with assignment of each carbons.

## Supplementary information 6

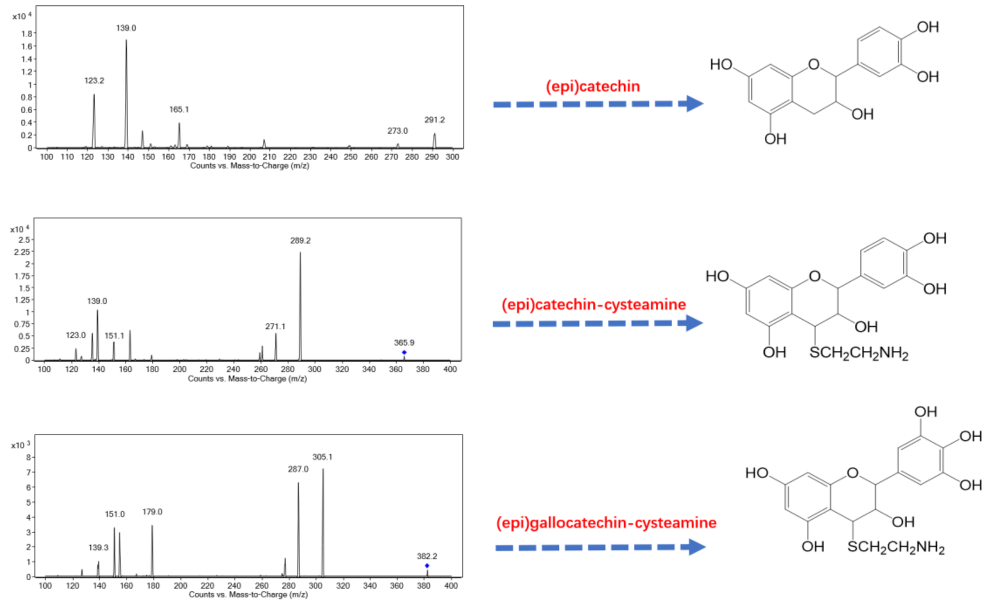


Figure S6. The spectrum of the subunits obtained by ESI-MS/MS analysis.

**Supplementary information 7**

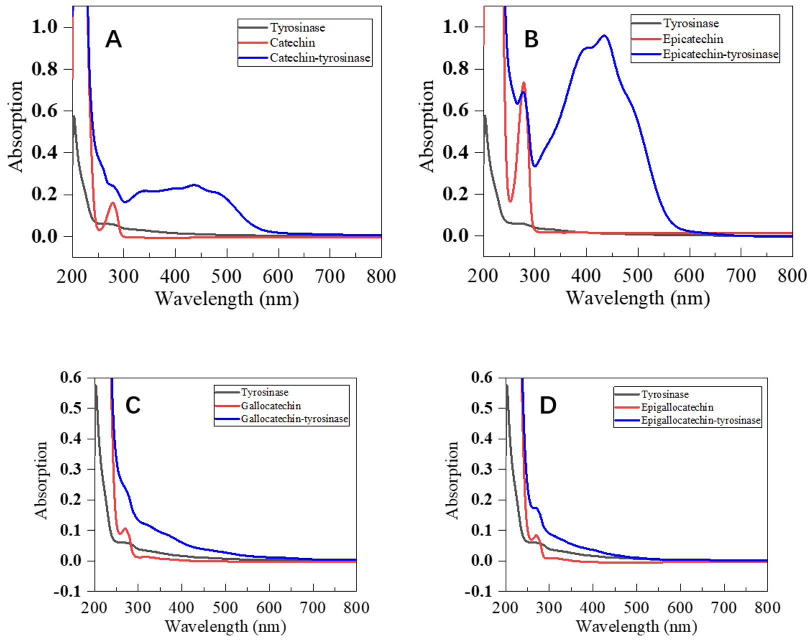


Figure S7. The UV-Vis spectra of 50 μg/mL catechin (A), 50 μg/mL epicatechin (B), 50 μg/mL gallocatechin (C), 50 μg/mL epigallocatechin (D) before (red curves) and after (blue curves) react with 1 mg/mL tyrosinase, referencing with the UV-Vis spectra of tyrosinase (1 mg/mL). The spectrum was taken after 10 min reaction in a 25 $^{o}$C water bath. The samples, including model compounds and tyrosinase were dissolved in 50 mM PBS solution.
